# Cortical morphology in children with alcohol-related neurodevelopmental disorder

**DOI:** 10.1002/brb3.191

**Published:** 2013-11-27

**Authors:** Meghna Rajaprakash, M Mallar Chakravarty, Jason P Lerch, Joanne Rovet

**Affiliations:** 1 Neurosciences and Mental Health Research Program, The Hospital for Sick ChildrenToronto, Ontario, Canada; 2 Faculty of Medicine, University of TorontoToronto, Ontario, Canada; 3 Department of Psychiatry, University of TorontoToronto, Ontario, Canada; 4 Institute of Biomaterials and Biomedical Engineering, University of TorontoToronto, Ontario, Canada; 5 Kimel Family Imaging-Genetics Research Laboratory, Research Imaging Centre, Centre for Addiction and Mental HealthToronto, Ontario, Canada; 6 Department of Medical Biophysics, University of TorontoToronto, Ontario, Canada

**Keywords:** ARND, cortical thickness, MRI, surface area

## Abstract

**Introduction:**

It is well established that individuals exposed to alcohol in utero have reduced cortical grey matter volumes. However, the candidate determinants of these reductions, cortical thickness (CT) and surface area (SA), have not been investigated exclusively in alcohol-related neurodevelopmental disorder (ARND), the most prevalent fetal alcohol spectrum disorder subgroup that lacks the characteristic facial dysmorphology.

**Methods:**

T1-weighted magnetic resonance imaging scans were obtained from 88 participants (8–16 years), 36 diagnosed with ARND and 52 typically developing controls. Scans were submitted to the CIVET pipeline (version 1.1.10). Deformable models were used to construct the inner white matter surfaces and pial surfaces from which CT and SA measures were derived. Group differences in cortical volume, CT, and SA were computed using a general linear model covaried for age, sex, and handedness.

**Results:**

Global cortical volume reductions in ARND did not reflect CT, which did not differ between groups. Instead, volume decreases were consistent with global SA reductions in bilateral frontal and temporal as well as right occipital regions. Local reductions in SA were observed in the right superior temporal gyrus and the right occipital-temporal region.

**Conclusion:**

Results suggest that in ARND, prenatal alcohol exposure perturbs global SA to a greater degree than CT, particularly in the right temporal lobe.

## Introduction

The teratogenic effects of alcohol on the brain are well-documented in the condition known as fetal alcohol spectrum disorder (FASD), which is the umbrella term to describe the variety of conditions arising from prenatal alcohol exposure. The most well-known condition is fetal alcohol syndrome (FAS), which is characterized by the symptom triad that includes a dysmorphic face, growth retardation, and diverse cognitive and behavioral impairments (Stratton et al. 1996). Two relatively less common disorders are partial FAS (pFAS) with some but not all of the FAS features and alcohol-related birth defects with only physical abnormalities. However, the most prevalent form of FASD is alcohol-related neurodevelopmental disorder (ARND; Stoler and Holmes 1999; Riley and McGee 2005), which involves only cognitive and behavioral features (Stratton et al. 1996; Chudley et al. 2005) and lacks any of the physical stigmata. Nevertheless, ARND still involves a high risk of poor outcome and significant challenges for families and educators and it poses a major public health burden (Chudley et al. 2007) and high cost to society (Lupton et al. 2004; Stade et al. 2006).

Because alcohol has neurotoxic effects on the brain throughout gestation, children with FASD show diverse brain abnormalities regardless of etiology. A large body of literature has substantiated global brain volume reductions (Archibald et al. 2001; Astley et al. 2009; Norman et al. 2009) with specific reductions in parietal, temporal, and frontal lobes (Sowell et al. 2002; Spadoni et al. 2007; Bjorkquist et al. 2010; for a review see Lebel et al. 2011), and in the caudate (Cortese et al. 2006), hippocampus (Riikonen et al. 1999, 2005; Autti-Ramo et al. 2002; Willoughby et al. 2008; Coles et al. 2011), and cerebellum (Sowell et al. 1996). Likewise, children with FASD show cortical and subcortical grey matter reductions (Astley et al. 2009; Nardelli et al. 2011), white matter abnormalities (Lebel et al. 2008; Wozniak et al. 2009), and structural abnormalities of the corpus callosum (Riley et al. 1995; Autti-Ramo et al. 2002). In most studies to date, samples have either comprised mixed FASD subgroups (Lebel et al. 2008; Wozniak et al. 2009) or directly compared individual subgroups (Astley et al. 2009). Few if any studies have examined the ARND subtype exclusively, despite its prevalence and recognition as a distinct neurodevelopmental disorder (NIAAA 2011). As children with ARND lack specific physical markers, diagnosis of this disorder is often difficult, especially given frequent comorbidities with other neurodevelopmental disorders and influences of other adverse events including poverty, neglect, and poor nutrition. Thus, brain biomarkers of prenatal alcohol exposure may facilitate diagnosis. One such biomarker is cortical morphology, which is shown to be abnormal in distinct brain regions in various other neurodevelopmental disorders (e.g., Almedia et al. 2010; Raznahan et al. 2010; Duerden et al. 2012), as well as FASD (e.g., Sowell et al. 2008).

Four studies have to date evaluated one aspect of cortical morphology, namely cortical thickness (CT), in individuals with FASD. However, these studies have produced inconsistent findings. Sowell et al. (2008), Fernández-Jaén et al. (2011), and Yang et al. (2012) reported increased CT in large regions of the temporal, parietal, and frontal lobes, whereas Zhou et al. (2011) described cortical thinning in similar regions. The explanations to account for these discrepancies may reflect the different patient-and control-recruitment methods, diagnostic approaches, participant characteristics, and magnetic resonance imaging (MRI) processing techniques among the studies. One factor that may also differentiate the divergent results is sample composition since the studies showing cortical thickening were comprised exclusively (Fernández-Jaén et al. 2011) or mostly of FAS cases (Sowell et al. 2008; Yang et al. 2012) and the study indicating cortical thinning had mostly non-FAS alcohol-exposed cases (Zhou et al. 2011). Another factor is the age ranges of the samples: Fernández-Jaén et al. (2011) and Yang et al. (2012) investigated 7–16 year olds, whereas Zhou et al. (2011) involved a much broader age range (6–30 years). This is relevant given the major changes in both cortical thickening and thinning that occur across this age range (Shaw et al. 2008).

From basic science, it is known that cortical surface area (SA) and CT represent two critical aspects of cortical morphology that differ in terms of their genetic origins (Panizzoni et al. 2009), cellular processes (Rackic 1995), and tempos of postnatal developmental change (Raznahan et al. 2011). It is thought that SA is mainly established in early embryogenesis when progenitor cells divide symmetrically at the ventricular zone (Chenn and Walsh 2002) to produce the founders of the ontogenetic radial columns that define the magnitude of cortical area (Mountcastle 1997). CT development is believed to occur later and arise from the asymmetric division of progenitor cells that migrate along radial glial cells to build the columns (Rackic 1995) at the cortical plate (Rackic 1978; Gadisseaux et al. 1990). Thus, if alcohol exposure occurs early in gestation, SA may be affected to a greater degree than CT. CT and SA can also be modified through postnatal influences that affect dendritic arborization and pruning processes (Huttenlocher 1990), intra-cortical myelination (Sowell et al. 2004; Geidd et al. 2008), and neuronal apoptosis of founder cells (Ikonomidou et al. 2000). Because SA and CT are both determinants of cortical volume, which is reduced in children with FASD, further investigation of all parameters (viz., SA, CT, cortical volume) may help elucidate how prenatal alcohol exposure affects cortical development.

This study on the ARND subtype exclusively was designed to evaluate which aspects of cortical morphometry indices are affected in these patients. We hypothesized that patients would show significantly reduced SA and CT given their smaller cortical volumes and similarity to the patients studied by Zhou et al. (2011). In light of findings showing that children with FASD have deficits in executive functioning, sensorimotor skills, and verbal and visual processing (Mattson et al. 1996; Rasmussen et al. 2006; Kodituwakku 2007), we also hypothesized their CT and SA abnormalities would be most evident in brain regions subserving these functions, namely frontal, temporal, and parietal lobes.

## Material and Methods

### Participants

Participants included 88 children ranging in age from 8.1 to 15.6 years. Thirty-six (17 males) had ARND and fifty-two (30 males) were typically developing controls, all of whom received MRI scans in a single scanner as part of several ongoing studies. Initial screening included lack of preterm birth, head injury, debilitating or chronic medical condition, and MRI contraindications such as braces and metal implants. Parents or caregivers provided written informed consent and participants orally assented to participate. Procedures for this study protocol were approved by the Research Ethics Board of the Hospital for Sick Children.

The ARND group (mean age = 11.4 years, range = 8.1–15.1 years) consisted of patients diagnosed previously at The Hospital for Sick Children Motherisk Follow-up Clinic, which serves as a regional diagnostic facility for FAS and ARND. Most children attending this clinic were accompanied by foster parents, adoptive parents, and/or caseworkers from the Children's Aid Society (CAS), while a minority came with a biological parent or relative. Clinic staff included: (i) a board certified pediatrician trained in FAS diagnosis who also performed neurological and physical assessments and assessed for facial dysmorphology and (ii) a registered psychologist, psychometrist, and speech therapist who performed different aspects of the comprehensive neuropsychological assessment children were given. Diagnoses were made using the Canadian guideline system (Chudley et al. 2005), which first and foremost requires documented evidence of substantial prenatal alcohol exposure ascertained from (a) foster, adoption, or CAS records indicating child was legally removed from mother due to her alcohol abuse during pregnancy or later neglect for alcoholism-related reasons, (b) reports from relatives assuming kinship care stating that they observed heavy maternal drinking during pregnancy, or (c) maternal self-report of heavy drinking during pregnancy. In the handful of adopted children without CAS substantiation, maternal drinking was assessed through extensive interview of the adoptive parents, who all were informed of heavy maternal drinking during pregnancy. To receive a diagnosis of ARND, a child had to show significant deficits in three distinct functional domains (e.g., attention, executive function, learning and memory, verbal processing) and not have either growth deficiency or facial dysmorphology (philtrum and palpebral fissure size both <10 percentile).

The control group consisted of typically developing children, who were former participants in previous studies and in the same age range as the ARND group (mean age = 11.0 years, age range = 8.2–15.6 years). Controls were originally recruited through community and hospital postings or were biological children of a participating adoptive or foster parent. None had a documented history of prenatal exposure to alcohol or other teratogenic substances, a learning disability, or other neurological or psychiatric condition.

### Demographics

Parents or caregivers completed a child history form that included comprehensive information of the child's prenatal, birth, developmental, and familial history. Socioeconomic status (SES) was computed using the Hollingshead Four-Factor Index (Hollingshead 1975) based on the education and occupation of biological or foster parents. All participants were assessed for intelligence with the Wechsler Abbreviated Scale of Intelligence (WASI; Wechsler 1999), which provides a full-scale IQ score.

### Image acquisition and processing

High-resolution T1-weighted MRI scans were obtained in the axial plane (repetition time = 10.06 msec, echo time = 4.2 msec, inversion time = 400 msec, flip angle = 20°, field of view = 180 mm, acquisition matrix = 256 × 192, slice thickness = 1.5 mm) using a 1.5 Tesla GE signal excite scanner (General Electric Medical Systems, Milwaukee, WI).

All scans were processed using the automated CIVET pipeline (version 1.1.10; Montreal Neurological Institute at McGill University, Montreal, Quebec, Canada). First, they were registered to the symmetric ICBM 152 template (Collins et al. 1994) and then corrected for radiofrequency inhomogeneity (Sled et al. 1998). Next, skulls were stripped from the brain tissue (Smith 2002), which was then classified into grey matter, white matter, and cerebrospinal fluid (CSF) components (Zijdenbos et al. 2002; Tohka et al. 2004). Deformable models were used to construct the inner white matter surface and grey matter–CSF interface or pial surface in both hemispheres (Kim et al. 2005). These yielded four surfaces of 40,962 vertex points per surface. CT was measured from each vertex point on the white matter surface to the corresponding pial-surface point (Lerch and Evans 2005). CT data were blurred with a 20-mm surface-based diffusion-blurring kernel (Chung and Taylor 2004) and nonlinearly aligned using surface-based registration (Lyttelton et al. 2007). SA was computed at each vertex point of the pial surface by estimating the two dimensional area of a triangle formed by three vertices on the surface mesh and attributing a third of this area to each of the three vertices (Lyttelton et al. 2009).

In addition to the vertex-wise analysis, each cortical hemisphere was segmented into sub-regions using the ANIMAL algorithm (Collins et al. 1995). From these data, measures of total cortical grey matter volume, total SA, and average CT were derived for each of the four hemispheric lobes (frontal, parietal, temporal, occipital), thus eight in total.

### Statistical analyses

The RMINC package (version 1.0) was used to analyze the vertex-wise data. Lobe-wise and hemispheric analyses were conducted using IBM SPSS Statistics 20.0 for Macintosh (Armonk, NY). Effect sizes were computed using Cohen's *d*, which indicates a small effect if between 0.2 and 0.3, a medium effect if around 0.5, and a large effect if between 0.8 and infinity.

Group differences in CT and SA were investigated at each vertex point using a general linear model controlling for age, gender, and handedness. Results were corrected for multiple comparisons using a False Discovery Rate set at 5%, whereby *q *<* *0.05 was significant (Genovese et al. 2002). Group differences in lobe-wise measures of cortical grey matter volume, SA, and average CT were assessed using a general linear model controlling for age, gender, and handedness. In order to eliminate variance associated with the global effects of prenatal alcohol exposure, cortical volume comparisons were corrected for total brain volume and SA analyses were covaried for total SA. To account for multiple comparisons from the eight lobar regions, lobe-wise results were corrected using the Bonferonni adjusted *α* level of 0.006 per test (0.05/8).

## Results

### Demographic and behavioral data

Table 1 shows demographic data for ARND and control groups. Groups did not differ in age, handedness, or gender. However, ARND had significantly lower IQ (*P *<* *0.001) and SES (*P *=* *0.002) than controls. A greater number of participants in the ARND group were in foster or adoptive care than controls. The ARND group was also more likely to have been exposed secondarily to cigarettes and other drugs and to have received a diagnosis of attention deficit hyperactivity disorder (ADHD) than children in the control group.

**Table 1:** Demographic information for ARND and control groups.

	ARND	Controls	*P-*value
Sample size	36	52	
Mean age (years)	11.4 (1.9)	11.0 (1.5)	ns
Gender (% M/F)	47.2/52.8	57.7/42.3	ns
Socioeconomic status 1(highest)–5(lowest)	1.73 (0.87)	2.47 (1.13)	*P* = 0.002
Mean full-scale IQ (SD)	89.6 (12.45)	114.9 (12.2)	*P* < 0.001
IQ range	60–116	75–143	
Handedness (% cases)			
Right	83.3	82.7	
Left	11.1	9.6	
Both	2.8	0	
Unspecified	2.8	7.7	
Participants in			
Biological care	7	52	
Foster care	2	0	
Adoptive care	27	0	
Participants with			
Secondary prenatal exposure to cigarettes	17	1	
Secondary prenatal exposure to marijuana	5	0	
Secondary prenatal exposure to cocaine	6	0	
Secondary prenatal exposure to unspecified drugs	11	0	
Diagnosis of ADHD	22	1	

In the ARND group, head circumference was below the 10th percentile in 4% of cases (mean percentile = 39.3, SD = 25.0, range = 5–90), none had a philtrum below the 10th percentile in length (mean percentile = 36.9, SD = 20.6, range = 10–75), and palpebral fissure length was below the 10th percentile in 8% of cases (mean percentile = 41.9, SD = 19.3). None of the cases with a small head circumference also had a small philtrum or palpebral fissure, while the few cases with short philtrums had very large head circumferences and normal palpebral fissure lengths.

### Brain volumes

**Figure 1:**
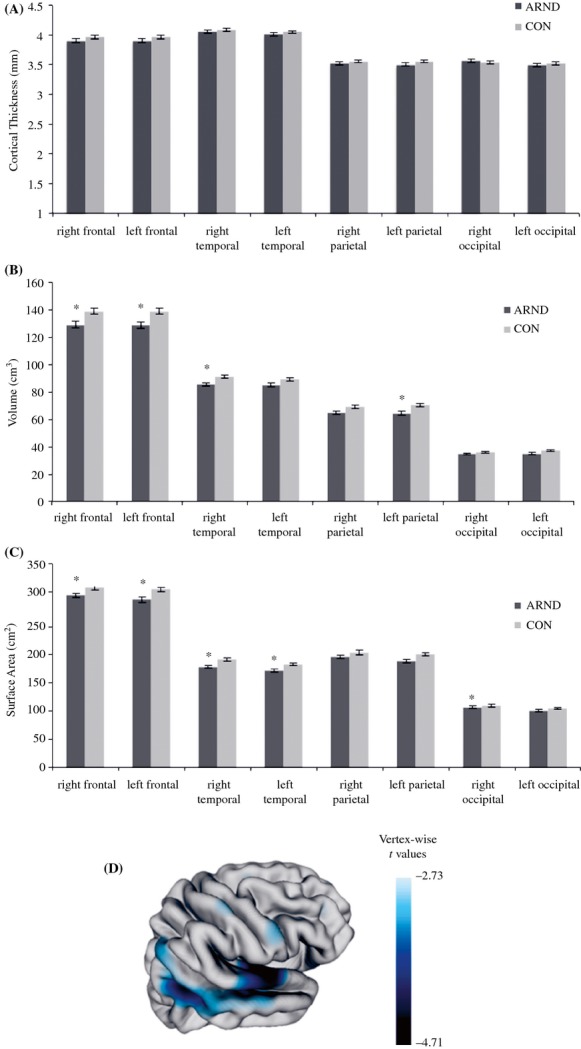
(A) Nonsignificant group differences in mean lobar cortical thickness values. (B) Uncorrected group differences in lobar cortical grey matter volumes after controlling for age, gender, and handedness (*Significant group difference at *P* < 0.006). (C) Uncorrected group differences in lobar surface area (SA) after controlling for age, gender, and handedness (*Significant group difference at *P* < 0.006). (D) Right hemisphere brain map portraying *t* values of group comparisons after correcting for multiple comparisons using FDR (*q* < 0.05). Blue colors indicate areas of significant SA reductions in ARND.

The ARND group showed significant reductions in total brain volumes (*F *=* *10.74, *P *=* *0.002, Cohen's *d *=* *0.80) and grey matter volumes (*F *=* *8.05, *P *=* *0.006, Cohen's *d *=* *0.77). Results uncorrected for total brain volume showed the ARND group had significantly smaller absolute volumes of left and right frontal (*P *=* *0.006, *P *=* *0.004), left parietal (*P *=* *0.003), and right temporal grey matter (*P *=* *0.004) than controls (see Fig. 1B). However, when we corrected for total cortical volume, none of the lobar cortical volume differences remained significant.

### Cortical thickness

Vertex-wise and lobe-wise analyses on uncorrected data as well as data corrected for multiple comparisons at 5% False Discovery Rate showed no significant group differences in CT within left or right hemispheres. Figure 1A presenting the overall mean CT values by group illustrates this effect.

### Surface area

Results uncorrected for total SA indicated that relative to controls, the ARND group had significant reductions in left and right frontal (*P *=* *0.005 and 0.002), left and right temporal (*P *=* *0.006 and 0.001), and right occipital (*P *=* *0.004) lobes (see Fig. 1C). The ARND group also showed a reduction in total SA (*F *=* *8.31, *P *=* *0.005 Cohen's *d *=* *0.73). However, when we controlled for this global effect, only the right temporal lobe SA approached significant, (*F *=* *3.86, *P *=* *0.05, Cohen's *d *=* *0.78). Further vertex-wise analyses revealed these SA abnormalities were confined to the right superior temporal gyrus and a region between the right temporal and occipital cortices, *t*(86) = −2.81, *q *<* *0.05 (see Fig. 1D).

### Age trajectories

No significant age by group interactions were found on measures of CT and SA at both the hemispheric and lobar levels. The main effects of age on total brain volume (*F *=* *2.27, *P *=* *0.09), total SA (*F *=* *2.56, *P *=* *0.32), and mean CT (*F *=* *1.45, *P *=* *0.59) were not significant.

## Discussion

This study aimed to determine whether children with ARND differed from typically developing controls in cortical morphometry measures. We observed global brain volume reductions in frontal, parietal, temporal cortical regions in the ARND group; however, these reductions did not reflect CT abnormalities as groups did not differ on this index. Instead, the ARND group showed significant cortical SA reductions in bilateral frontal and temporal and right occipital regions and after we controlled for global effects, local reductions in SA of the right temporal lobe approached significance. Vertex-wise analyses also revealed these SA reductions were confined to the right superior temporal gyrus and the right occipital-temporal area.

Our findings concur with past research showing that fetal-alcohol-affected individuals have global grey matter volume reductions in frontal, temporal, and parietal lobes. In addition, as observed in previous studies (Mattson et al. 1994; Archibald et al. 2001; Bjorkquist et al. 2010), the effects did not remain significant when we corrected for total brain volume. The current findings also parallel past research that showed reduced surface extent in FASD, particularly in the orbitofrontal regions (Sowell et al. 2002).

Surprisingly, our CT results failed to differentiate individuals with ARND from controls, thus providing no support for previous studies showing either cortical thinning (Zhou et al. 2011) or cortical thickening (Sowell et al. 2008; Fernández-Jaén et al. 2011; Yang et al. 2012) in areas of the frontal, temporal, and parietal cortices. This lack of difference may reflect methodological differences in the various image processing pipelines used and the methods for correcting for multiple comparisons, as well as the different sample compositions across studies that varied in diagnoses, age ranges, and levels and timing of prenatal alcohol exposure. Indeed, previous studies have shown cortical thickening when samples had a greater preponderance of cases at the FAS-end of the spectrum (Sowell et al. 2008) and cortical thinning when a greater proportion of non-FAS alcohol-exposed cases were included (Zhou et al. 2011). While this study sought to eliminate this variability by focusing strictly on the most prevalent ARND subgroup, our participants may also have varied among themselves as to the severity of their neurobehavioral symptoms. Thus, it is possible that our lack of effect reflected some severely affected participants showing cortical thickening and others showing thinning of the cortex. It should be noted, however, that our cases were likely more severely affected than those in the Zhou et al. (2011) as some of the participants described in that study would not have achieved diagnosis in our clinic (Nash et al. 2013).

Several of the previous studies involved a broad age range that extended into adulthood (e.g., Sowell et al. 2008; Zhou et al. 2011). Our sample consisted primarily of young participants, the majority of whom were between 10 and 12 years of age at time of scanning. As such, our results are representative of the ARND clinical group at a circumscribed developmental stage, which reflects the pre-to early adolescent period primarily. This is critical for interpreting present findings because CT has been shown to vary in a curvilinear manner with age reflecting a preadolescent increase followed by a postadolescent decrease (Shaw et al. 2008). Our, our lack of effect may have reflected the fact that our sample included both participants whose cortices were in the process of thickening as well as those who were in the preliminary later stages of thinning. In contrast, the Zhou et al. (2011) findings of thinning may have reflected a disproportionate number of older participants showing thinning of the cortex.

Our observation of SA but not CT abnormalities in children with ARND is, to our knowledge, novel. In view of basic research findings showing a dissociation between these measures in terms of timing (Rackic 1995) with SA emanating earlier from symmetrical division of progenitor cells in the periventricular zone (Chenn and Walsh 2002) and CT from asymmetrical division later, our participants may have been exposed to alcohol early in gestation. Although facial dysmorphology (which our sample lacked) is usually associated with very early exposure (Anthony et al. 2010), it is possible that the participants in this study were not exposed during the precise period for the relevant features to form fully (Suttie et al. 2013).

The results of this study also showed local effects with abnormalities in a region between the right temporal and right occipital cortices. Previous studies have also indicated that individuals exposed prenatally to alcohol have structural grey matter volume reductions in the occipital-temporal area (Sowell et al. 2002; Li et al. 2008), which is implicated in visual processing, specifically for the recognition of object features (Beauchamp 2005) and is strongly governed by attention processes (Kanwisher and Wojciulik 2000). Accordingly, Li et al. (2008) found that when individuals with prenatal alcohol exposure performed a sustained visual attention tasks involving shape recognition, they exhibited functional abnormalities in this area.

The other brain region differentiating groups was the right superior temporal gyrus, which is important for social cognition (Baron-Cohen et al. 1999) and is abnormal in individuals with autism (Jou et al. 2010). Autopsy findings by Casanova et al. (2002) demonstrating that the cell columns defining SA in the posterior superior temporal gyrus were significantly smaller in cases with autism has potential relevance for the social cognition deficits in ARND (Greenbaum et al. 2009) as groups show similar socially inappropriate behaviors (Bishop et al. 2007; Stevens et al. 2012). Other functions of the right superior temporal gyrus include auditory discrimination (Bueti et al. 2008), given close proximity to the auditory cortex, and spatial orienting to gaze cues (Akiyama et al. 2006), which are also problematic in individuals with FASD.

Although current results provide novel insights on the cortical abnormalities of patients diagnosed with ARND, several limitations warrant further discussion. First, as our sample was ascertained retrospectively through a clinic, we could not obtain precise measurement of the actual dose or timing of the exposure. Nonetheless, degree of alcohol exposure was well-described in cases ascertained through the CAS and testaments of mothers or relatives usually indicated a large volume of alcohol had been consumed. For example, grandparents and other relatives (e.g., aunts, sisters-in-law), who represent a substantial kinship group that serve as caregivers to a related child, have described very heavy drinking throughout gestation including at the end of pregnancy. Also, many of the foster or adopted children were taken at birth from their mothers due to her heavy drinking throughout pregnancy. Second, as is typical in FASD clinic-based studies, it was not possible to control for confounding environmental factors such as poor pregnancy care, early life adversity, poverty, prenatal exposure to cigarettes and other drugs, stress, multiple home placements, and neglect abuse, all of which profoundly influence the developing cortex (Abel and Hannigan 1995; Sowell et al. 2008; Toro et al. 2008). Third, regarding our ARND sample, we found they differed significantly from controls in IQ and were much more likely to have comorbidities such as ADHD that are associated with cortical abnormalities (Fernández-Jaén et al. 2011). However, follow-up analyses showed the results did not differ when children with an IQ below 70 were excluded and when we compared those with ARND and ADHD from those without the ADHD diagnosis. Comparable analysis of other affiliated comorbidities (e.g., autism, conduct disorder) was not conducted. Furthermore, since the diagnostic criteria of the Canadian system (Chudley et al. 2005) are broadly defined, it is possible the ARND group in this study represented a quite heterogeneous group of children, thus contributing to the lack of effect in CT. Lastly, increased movement in the ARND group versus controls may have also introduced some biases.

## Conclusion

Global cortical volume reductions seen in children and young adolescents with ARND were shown to reflect global SA reductions, particularly in the right temporal lobe and especially the occipital-temporal area and superior temporal gyrus, but not cortical thinning or thickening. Further research is needed to elucidate the specific nature and sustainability of the observed SA abnormalities in samples of different ages and other forms of FASD to ascertain whether these foci are pathognomic. Research is also needed to determine the implications of current findings for cognitive functioning in children with ARND.

## References

[b1] Abel EL, Hannigan JH (1995). Maternal risk factors in fetal alcohol syndrome: provocative and permissive influences. Neurotoxicol. Teratol.

[b2] Akiyama T, Kato M, Muramatsu T, Saito F, Umeda S, Kashima H (2006). Gaze but not arrows: a dissociative impairment after right superior temporal gyrus damage. Neuropsychologia.

[b3] Almedia LG, Ricardo-Garcell J, Prado H, Barajas L, Fernández-Bouzas A, Ávila D (2010). Reduced right frontal cortical thickness in children, adolescents, and adults with ADHD and its correlation to clinical variables: a cross-sectional study. J. Psychiatr. Res.

[b5] Anthony B, Vinci-Booher S, Wetherill L, Ward R, Goodlett C, Zhou FC (2010). Alcohol-induced facial dysmorphology in C57BL/6 mouse models of fetal alcohol spectrum disorder. Alcohol.

[b6] Archibald SL, Fennema-Notestine C, Gamst A, Riley EP, Mattson SN, Jernigan TL (2001). Brain dysmorphology in individuals with severe prenatal alcohol exposure. Dev. Med. Child Neurol.

[b7] Astley SJ, Aylward EH, Olson HC, Chen X, Kern K, Brooks A (2009). Magnetic resonance outcomes from a comprehensive magnetic resonance imaging study of children with fetal alcohol spectrum disorders. Alcohol. Clin. Exp. Res.

[b8] Autti-Ramo I, Autti T, Korkman M, Kettunen S, Salonen O, Valanne L (2002). MRI findings in children with school problems who had been exposed prenatally to alcohol. Devel. Med. Child Neurol.

[b9] Baron-Cohen S, Ring HA, Wheelwright S, Bullmore ET, Brammer MJ, Simmons A (1999). Social intelligence in the normal and autistic brain: an fMRI study. Eur. J. Neurosci.

[b10] Beauchamp MS (2005). See me, hear me, touch me: multisensory integration in lateral occipital-temporal cortex. Curr. Opin. Neurobiol.

[b11] Bishop S, Gahagan S, Lord C (2007). Re-examining the core features of autism: a comparison of autism spectrum disorder and fetal alcohol spectrum disorder. J. Child Psychol. Psychiatry.

[b12] Bjorkquist OA, Fryer SL, Reiss AL, Mattson SN, Riley EP (2010). Cingulate gyrus morphology in children and adolescents with fetal alcohol spectrum disorders. Psychiatry Res.

[b13] Bueti D, Van Dongan EV, Walsh V (2008). Superior temporal cortex in auditory timing. PLoS ONE.

[b14] Casanova MF, Buxhoeveden DP, Switala AE, Roy E (2002). Minicolumnar pathology in autism. Neurology.

[b15] Chenn A, Walsh CA (2002). Regulation of cerebral cortex size by control of cell cycle exit in neuronal precursors. Science.

[b16] Chudley AE, Conry J, Cook JL, Loock C, Rosales T, LeBlanc N (2005). Fetal alcohol spectrum disorder: Canadian guidelines for diagnosis. Canad. Med. Assoc. J.

[b17] Chudley AE, Kilgour AR, Cranston M, Edwards M (2007). Challenges of diagnosis in fetal alcohol syndrome and fetal alcohol spectrum disorder in the adult. Am. J. Med. Genet. C Semin. Med. Genet.

[b18] Chung MK, Taylor J (2004). Diffusion smoothing on brain surface via finite element method. Proceedings of Biomedical Imaging: Macro to Nano, IEEE International Symposium.

[b19] Coles CD, Goldstein FC, Lynch ME, Chen X, Kable JA, Johnson KC (2011). Memory and brain volume in adults prenatally exposed to alcohol. Brain Cogn.

[b20] Collins DL, Neelin P, Peters TM, Evans AC (1994). Automatic 3-D intersubject registration of MR volumetric data in standard talairach space. J. Comp. Assist. Tomogr.

[b21] Collins DL, Holmes CJ, Peters TM, Evans AC (1995). Automatic 3-D model-based neuroanatomical segmentation. Hum. Brain Mapp.

[b22] Cortese BM, Moore GJ, Bailey BA, Jacobson SW, Delaney-Black V, Hannigan JH (2006). Magnetic resonance and spectroscopic imaging in prenatal alcohol-exposed children: preliminary findings in the caudate nucleus. Neurotoxicol. Teratol.

[b23] Duerden EG, Tannock R, Dockstader C (2012). Altered cortical morphology in sensorimotor processing regions in adolescents and adults with attention-deficit/hyperactivity disorder. Brain Res.

[b24] Fernández-Jaén A, Fernandes-Mayoralas DM, Tapia DQ, Calleja-Perez B, Garcia-Segura JM, Arribas SL (2011). Cortical thickness in fetal alcohol syndrome and attention deficit disorder. Pediatr. Neurol.

[b26] Gadisseaux J-F, Kadhim HJ, Caviness P, Van den Bosch de Aguilar VS, Evard P (1990). Neuron migration within the radial glial fiber system of the developing mutine cerebrum: an electron microscopic autoradiographic analysis. Brain Res. Dev. Brain Res.

[b27] Geidd JN, Keshavan M, Paus T (2008). Why do many psychiatric disorders emerge during adolescence?. Nat. Rev. Neurosci.

[b28] Genovese CR, Lazar NA, Nicols T (2002). Thresholding of statistical maps in functional neuroimaging using the false discovery rate. Neuroimage.

[b29] Greenbaum RL, Stevens SA, Nash K, Koren G, Rovet J (2009). Social cognitive and emotion processing abilities of children with fetal alcohol spectrum disorders: a comparison with attention deficit hyperactivity disorder. Alcohol. Clin. Exp. Res.

[b30] Hollingshead A (1975). Four factor index of social status.

[b31] Huttenlocher PR (1990). Morphometric study of human cerebral cortex development. Neuropsychologia.

[b32] Ikonomidou C, Bittigau P, Ishimaru MJ, Wozniak DF, Koch C, Genz K (2000). Ethanol-induced apoptotic neurodegeneration and fetal alcohol syndrome. Science.

[b33] Jou RJ, Minshew NJ, Keshavan MS, Vitale MP, Hardan AY (2010). Enlarged right superior temporal gyrus in children and adolescents with autism. Brain Res.

[b34] Kanwisher N, Wojciulik E (2000). Visual attention: insights from brain imaging. Nat. Rev. Neurosci.

[b35] Kim JS, Singh V, Lee JK, Lerch J, Ad-Dab'bagh Y, MacDonald D (2005). Automated 3-D extraction and evaluation of the inner and outer cortical surfaces using a laplacian map and partial volume effect classification. Neuroimage.

[b36] Kodituwakku PW (2007). Defining the behavioural phenotype in children with fetal alcohol spectrum disorders: a review. Neurosci. Behav. Rev.

[b37] Lebel C, Rasmussen C, Wyper K, Walker L, Andrew G, Yager J (2008). Brain diffusion abnormalities in children with fetal alcohol spectrum disorder. Alcohol Clin. Exp. Res.

[b38] Lebel C, Rousette F, Sowell ER (2011). Imaging the impact of prenatal alcohol exposure on the structure of the developing human brain. Neuropsychol. Rev.

[b39] Lerch JP, Evans AC (2005). Cortical thickness analysis examined through power analysis and a population simulation. Neuroimage.

[b40] Li Z, Coles CC, Lynch ME (2008). Occipital-temporal reduction and sustained visual attention deficit in prenatal alcohol exposed adults. Brain Imag. Behav.

[b41] Lupton C, Burd L, Harwood R (2004). Cost of fetal alcohol spectrum disorders. Am. J. Med. Genet. C Semin. Med. Genet.

[b42] Lyttelton O, Boucher M, Robbins S, Evans AC (2007). An unbiased iterative group registration template for cortical surface analysis. Neuroimage.

[b43] Lyttelton O, Karama S, Ad-Dab'bah Y, Zatorre RJ, Carbonell F, Worsley K (2009). Positional and surface area asymmetry of the human cortex. Neuroimage.

[b100] Mattson SN, Riley EP, Jernigan TL, Garcia A, Kaneko WM, Ehlers CL (1994). A decrease in the size of the basal ganglia following prenatal alcohol exposure: a preliminary report. Neurotoxicol. Teratol.

[b44] Mattson SN, Riley EP, Delis DC, Stern C, Lyons K (1996). Verbal learning and memory in children with fetal alcohol syndrome. Alcohol. Clin. Exp. Res.

[b45] Mountcastle V (1997). The columnar organization of the neocortex. Brain.

[b46] Nardelli A, Lebel C, Rasmussen C, Andrew G, Beaulieu C (2011). Extensive deep gray matter volume reductions in children and adolescents with fetal alcohol spectrum disorder. Alcohol. Clin. Exp. Res.

[b47] Nash K, Stevens S, Rovet J, Fantus E, Nulman I, Sorbara D (2013). Towards identifying a characteristic neuropsychological profile for fetal alcohol spectrum disorders. 1. Analysis of the motherisk FASD Clinic. J. Popul. Ther. Clin. Pharmacol.

[b48] NIAAA (2011). Consensus statement on recognizing alcohol-related neurodevelopmental disorder (ARND) in primary health care of children.

[b49] Norman AL, Crocker N, Mattson SN, Riley EP (2009). Neuroimaging and fetal alcohol spectrum disorders. Dev. Disab. Res. Rev.

[b50] Panizzoni CF-N, Eyer L, Jernigan T, Prom-Wormley E, Neale M, Jacobsen K (2009). Distinct genetic influence on cortical surface area and cortical thickness. Cereb. Cortex.

[b51] Rackic P (1978). Neuronal migration and contact guidance in the primate telencephalon. Postgrad. Med. J.

[b52] Rackic P (1995). A small step for the cell, a giant leap for mankind: a hypothesis of neocortical expansion during evolution. Trends Neurosci.

[b53] Rasmussen C, Horne K, Witol A (2006). Neurobehavioral functioning in children with fetal alcohol spectrum disorder. Child Neuropsychol.

[b54] Raznahan A, Toro R, Daly E, Robertson D, Murphy C, Deely Q (2010). Cortical anatomy in autism spectrum disorder: an in vivo MRI study on the effect of age. Cereb. Cortex.

[b55] Raznahan A, Shaw P, Lalonde F, Stockman M, Wallace GL, Greenstein D (2011). How does your cortex grow?. J. Neurosci.

[b56] Riikonen RS, Salonen I, Partanen K, Verho S (1999). Brain perfusion SPECT and MRI in fetal alcohol syndrome. Dev. Med. Child Neurol.

[b57] Riikonen RS, Nokelainen P, Valkonen K, Kolehmainen AI, Kumpulainen KI, Könönen M (2005). Deep serotonergic and dopaminergic structures in fetal alcoholic syndrome: a study with nor-b-CIT-single photon emission computed tomography and magnetic resonance imaging volumetry. Biol. Psychiatry.

[b58] Riley EP, McGee CL (2005). Fetal alcohol spectrum disorders: an overview with emphasis on changes in brain and behavior. Exp. Biol. Med.

[b59] Riley EP, Mattson SN, Sowell ER, Jernigan TL, Sobel DF, Jones KL (1995). Abnormalities of the corpus callosum in children prenatally exposed to alcohol. Alcohol Clin. Exp. Res.

[b60] Shaw P, Kabani NJ, Lerch JP, Eckstrand K, Lenroot R, Gogtay N (2008). Neurodevelopmental trajectories of the human cerebral cortex. J. Neurosci.

[b61] Sled JG, Zijdenbos AP, Evans AC (1998). A nonparametric method for automatic correction of intensity nonuniformity in MRI data. IEEE Trans. Med. Imaging.

[b62] Smith SM (2002). Fast robust automated brain extraction. Hum. Brain Mapp.

[b63] Sowell ER, Jernigan TL, Mattson SN, Riley EP, Sobel DF, Jones KL (1996). Abnormal development of the cerebellar vermis in children prenatally exposed to alcohol: size reduction in lobules I-V. Alcohol. Clin. Exp. Res.

[b64] Sowell ER, Thompson PM, Peterson BS, Mattson SN, Welcome SE, Henkenius AL (2002). Mapping cortical gray matter asymmetry patterns in adolescents with heavy prenatal alcohol exposure. Neuroimage.

[b65] Sowell ER, Thompson PM, Leonard CM, Welcome SE, Kan E, Toga AW (2004). Longitudinal mapping of cortical thickness and brain growth in normal children. J. Neurosci.

[b66] Sowell ER, Mattson SN, Kan E, Thompson PM, Riley EP, Toga AW (2008). Abnormal cortical thickness and brain-behavior correlation patterns in individuals with heavy prenatal alcohol exposure. Cereb. Cortex.

[b67] Spadoni AD, McGee CL, Fryer SL, Riley EP (2007). Neuroimaging and fetal alcohol spectrum disorders. Neurosci. Biobehav. Rev.

[b68] Stade B, Ungar W, Stevens B, Beyene J, Koren G (2006). The burden of prenatal exposure to alcohol: measurement of costs. J. FAS Intern.

[b69] Stevens SA, Nash K, Koren G, Rovet J (2012). Autism characteristics in children with fetal alcohol spectrum disorders. Child Neuropsychol.

[b70] Stoler M, Holmes LB (1999). Underrecognition of prenatal alcohol effects in infants of known alcohol abusing women. J. Pediatr.

[b71] Stratton KR, Howe CJ, Battaglia FC (1996). Fetal alcohol syndrome: diagnosis, epidemiology, prevention, and treatment.

[b72] Suttie M, Fouroud T, Wetherill L, Jacobson JL, Molteno CD, Meintjes EM (2013). Facial dysmorphism acress the fetal alcohol spectrum. Pediatrics.

[b73] Tohka J, Zijdenbos A, Evans A (2004). Fast and robust parameter estimation for statistical partial volume models in brain MRI. Neuroimage.

[b74] Toro R, Leonard G, Lerner JV, Lerner RM, Perron M, Pike GB (2008). Prenatal exposure to maternal cigarette smoking and the adolescent cerebral cortex. Neuropsychopharmacology.

[b75] Wechsler D (1999). Weschler abbreviated scale of intelligence.

[b76] Willoughby KA, Sheard ED, Nash K, Rovet J (2008). Effects of prenatal alcohol exposure on hippocampal volume, verbal learning, and verbal and spatial recall in late childhood. J. Int. Neuropsychol. Soc.

[b77] Wozniak JR, Muetzel RL, Mueller BA, McGee CL, Freerks MA, Ward EE (2009). Microstructural corpus callosum anomalies in children with prenatal alcohol exposure: an extension of previous diffusion tensor imaging findings. Alcohol. Clin. Exp. Res.

[b78] Yang Y, Roussette F, Kan E, Sulik KK, Mattson SN, Riley EP (2012). Abnormal cortical thickness alterations in fetal alcohol spectrum disorders and their relationships with facial dysmorphology. Cereb. Cortex.

[b79] Zhou D, Lebel C, Lepage C, Rasmussen C, Evans A, Wyper K (2011). Developmental cortical thinning in fetal alcohol spectrum disorders. Neuroimage.

[b80] Zijdenbos AP, Forghani R, Evans A (2002). Automatic “pipeline” analysis of 3D MRI data for clinical trials: application to multiple sclerosis. IEEE Trans. Med. Imaging.

